# DNA-Based Bacterial Community Profiles in Air-Dried Historical Soil Archives Are More Representative than Those from Rewetted Soils

**DOI:** 10.3390/microorganisms14030595

**Published:** 2026-03-06

**Authors:** Peng Lu, Bingjie Ji, Yuan Yan, Shulan Zhang, Xueyun Yang

**Affiliations:** 1 Shijiazhuang Institute of Pomology, Hebei Academy of Agricultural and Forestry Sciences, Shijiazhuang 050061, China; lupeng8602@163.com; 2 College of Natural Resources and Environment, Northwest A&F University, Yangling, Xianyang 712100, China; jibingjie2707@163.com (B.J.); 18812670037@163.com (Y.Y.); zhangshulan@nwafu.edu.cn (S.Z.); 3 Beijing Changping Soil Quality National Observation and Research Station, Changping, Beijing 100081, China

**Keywords:** temporal dynamics, loess soil, MiSeq sequencing, microbial features, relative abundance

## Abstract

Recording and tracking the long-term dynamic changes in microbial populations is as essential as monitoring other soil properties for evaluating soil quality and health; however, this area has significantly lagged due to technical constraints and challenges in storing fresh soil samples. Historically archived soil samples offer a unique opportunity to characterize the temporal dynamics of microorganisms over several decades. To determine whether archived air-dried soils can be utilized for this purpose, we compared the structure and composition of bacterial communities across fresh soils, air-dried soil archives stored for varying durations, and their corresponding rewetted counterparts, all sourced from a long-term fertilization experiment on calcareous loess soil. Soil microbial features were characterized using the MiSeq sequencing platform. The results indicated that the similarity of DNA-based bacterial community composition between fresh soil and both archived and rewetted soils followed a downward quadratic curve as archiving time increased. Specifically, the DNA-based community structure of soils air-dried and preserved for one year, as well as those rewetted after eight years of archiving, remained highly similar to that of fresh soil. Regarding taxonomic shifts, the relative abundance of *Actinobacteria* in both air-dried and rewetted soils increased with storage time. Conversely, the relative abundances of *Acidobacteria* and *Gemmatimonadetes* significantly increased in air-dried soils but decreased upon rewetting over time. The relative abundances of *Chloroflexi* and *Firmicutes* remained stable in air-dried soils; however, after rewetting, the former decreased while the latter increased dramatically. Furthermore, *Proteobacteria*, *Rokubacteria*, *Planctomycetes*, *Bacteroidetes*, and *Latescibacteria* exhibited a decreasing trend in both air-dried and rewetted soils. These findings suggest that air-dried soils preserve DNA-based community profiles more effectively than rewetted soils, particularly for samples stored for less than eight years. This study provides a valuable reference for utilizing archived historical soil samples from long-term experiments to investigate microbial community evolution.

## 1. Introduction

Microorganisms are widely recognized as one of the most important indicators of soil environmental conditions and are commonly used to assess soil quality and health [1,2]. They play a crucial role in maintaining ecosystem functions [3]. The abundance, diversity, and DNA-based community structure of soil microorganisms are highly sensitive to soil type and changes in environmental conditions, such as temperature, soil moisture, and tillage practices [4], with soil moisture being a particularly influential factor [5]. Consequently, fresh soil samples are generally preferred for evaluating the status of soil microbial communities. Soil microorganisms respond dynamically to variations in multiple soil properties, many of which change slowly over time. Therefore, elucidating the temporal dynamics of microbial communities in fresh soils requires long-term sampling and continuous measurement. However, during extended monitoring periods, analytical methods for microbial characterization have evolved substantially. For example, early techniques such as terminal restriction fragment length polymorphism (T-RFLP) and PCR-denaturing gradient gel electrophoresis (PCR-DGGE) have gradually been replaced by high-throughput MiSeq sequencing, which offers improved efficiency and accuracy. Methodological differences can inevitably introduce inconsistencies in the detected abundance and composition of microbial communities, making it difficult to draw reliable long-term conclusions. In addition, the long-term preservation of large quantities of fresh soil samples is often impractical due to storage constraints and high associated costs. As a result, most archived soil samples from long-term field experiments worldwide are air-dried [6]. These archived air-dried soils provide a potentially valuable resource for reconstructing the temporal dynamics of soil microbial communities over decadal or even centennial timescales [7]. Nevertheless, both the air-drying process and prolonged storage may profoundly affect microbial survival, leading to losses in microbial activity and genetic information and consequently altering the measured microbial diversity [8]. Therefore, it is essential to critically evaluate the feasibility and reliability of using archived air-dried soils for soil microbial research.

Several previous studies have evaluated the feasibility of using archived air-dried soils for microbial investigations [8,9,10]. For instance, Wang et al. (2021) [10] compared DNA-based bacterial community composition and structure between fresh soils and air-dried soils preserved for different durations (within one year) in a fluvo-aquic soil using Illumina sequencing. Their results indicated that both the composition and structure of bacterial communities in air-dried soils were highly similar to those observed in fresh soils. Similarly, Clark and Hirsch (2008) [9] investigated microbial abundance in fresh soils, freshly air-dried soils, and air-dried soils stored for 1, 59, and 89 years, all collected from a long-term fertilization experiment receiving either synthetic nitrogen, phosphate, and potassium fertilizers (NPK) or farmyard manure (FYM). Using denaturing gradient gel electrophoresis (DGGE), they found that bacterial abundance in soils preserved for one year under both NPK and FYM treatments did not differ significantly from that in the corresponding fresh soils. In contrast, soils preserved for 59 and 89 years exhibited a marked reduction in bacterial abundance relative to fresh soils. Notably, distinct fertilization effects on microbial preservation were observed during long-term storage: bacterial abundance in FYM-treated soils remained more comparable to that of fresh soils than in NPK-treated soils, suggesting a higher survival rate of bacteria under FYM management during the archiving process. The authors attributed this phenomenon to the protective effect of increased soil organic matter associated with FYM application [9]. Using 16S rRNA gene sequencing, Liu et al. (2019) [8] compared bacterial community composition and structure in eight typical arable soils archived for 1, 6, and 11 years across five ecological regions in China. Their results demonstrated that substantial microbial information could still be retrieved from archived soils, and that bacterial community composition changed only slightly over the 11-year storage period across all soil types. Collectively, these studies suggest that archived air-dried soils from long-term fertilization experiments can still be used to investigate microbial community dynamics and evolution, despite inevitable losses of microbial activity and genetic information during storage. However, some studies have argued that rewetting air-dried soils may partially restore microbial activity and thereby yield more reliable measurements. For example, Zhao et al. (2011) [11] reported that fluorescein diacetate (FDA) hydrolytic activity in a fluvo-aquic soil decreased to 14–40% of that in fresh soil after seven months of air-drying, but recovered to approximately 72% following 15 days of rewetting. Likewise, in a California grassland study, Barnard et al. (2014) [12] found that the abundance of potentially active bacteria (based on RNA analyses) declined during prolonged summer drought. Upon simulated rainfall, however, transcript copy numbers of the bacterial rpoB gene rapidly increased as soil moisture was restored, eventually exceeding pre-drought levels, indicating a rapid recovery of bacterial activity.

Nobili et al. (2006) [13] compared soil microbial activity, expressed as adenosine 5′-triphosphate (ATP), between fresh soils and rewetted (12-day incubation) air-dried soils archived for 14, 32, 50, and 83 years under NPK and farmyard manure (FYM) treatments in the Rothamsted Broadbalk experiment. Their results showed that ATP levels in rewetted soils from the NPK treatment were significantly lower than those in the corresponding fresh soils, declined progressively with increasing storage duration, and stabilized at approximately 5.0–5.4% of fresh soil values after more than 32 years of archiving. In contrast, ATP levels in FYM-treated soils archived for 14, 32, and 50 years remained at approximately 20.1–22.7% of those in fresh soils, but decreased to 10.5% after 83 years of storage. The authors attributed these contrasting responses between NPK and FYM treatments to differences in soil organic matter content, whereby higher carbon availability in FYM-amended soils provided greater protection and facilitated faster recovery of microbial activity upon rewetting [9,14]. Consequently, microbial activity in rewetted archived soils was strongly influenced by both fertilization regime and storage duration. In addition, Nobili et al. (2006) [13] compared ATP levels among fresh soils, freshly air-dried soils, and soils immediately rewetted after air-drying. They found that ATP concentrations decreased by 2.0–6.6-fold following air-drying and by 0.97–2.02-fold after immediate rewetting, relative to fresh soils. Nevertheless, owing to variations in soil organic carbon and nutrient availability resulting from different soil management practices, as well as differences in archiving duration, the combined effects of long-term storage and rewetting on microbial activity and community characteristics remain insufficiently understood. Therefore, in the present study, we compared DNA-based bacterial community composition and structure in fresh soils, air-dried soils archived for different durations (1, 8, 18, and 28 years), and their corresponding rewetted soils from an Anthrosol subjected to balanced and unbalanced synthetic fertilization in the Loess Plateau. By linking these treatments to differences in organic matter and their potential protective role for DNA in the Loess Plateau, this study aims to evaluate the potential protective role of organic matter for microbial DNA during long-term archiving and to assess whether air-dried historical soil archives can be reliably used to characterize microbial temporal dynamics over decadal timescales.

## 2. Materials and Methods

### 2.1. Study Site Description

The long-term field experiment was initiated in October 1990 under a winter wheat (*Triticum aestivum* L.)–summer maize (*Zea mays* L.) double-cropping system at the Chinese National Soil Fertility and Fertilizer Efficiency Monitoring Base for Loessial Soils (34°17′51″ N, 108°00′48″ E; 524.7 m a.s.l.), located in Yangling, Shaanxi Province, P. R. China. The soil at the site is a silt clay loam (16% clay, 52% silt, and 32% sand) derived from loess deposits and is classified as an Anthrosol according to the World Reference Base for Soil Resources [15]. The experimental site has a mean annual temperature of approximately 13.0 °C and a mean annual precipitation of 550 mm, with the majority of rainfall occurring between June and September. At the initiation of the experiment, the chemical properties of the arable soil layer (0–20 cm) were as follows: organic carbon, 7.44 g kg^−1^; total nitrogen, 0.93 g kg^−1^; Olsen phosphorus, 9.57 mg kg^−1^; exchangeable potassium, 191 mg kg^−1^; and soil pH, 8.62 (1:1 soil-to-water ratio) [16]. The long-term field experiment comprised 11 fertilization treatments, each established in a single plot (14 × 14 m^2^). For practical reasons, this experimental design inevitably introduced pseudoreplication, thereby limiting the strength of statistical inference. This constraint should be taken into account when interpreting treatment effects. Baseline soil fertility parameters, determined from composite samples collected across all plots prior to treatment implementation, exhibited low spatial variability (coefficient of variation ≤6% for most variables, except Olsen-P), likely reflecting decades of uniform fertilizer management and cropping practices followed by homogenized tillage. Comparable background conditions and experimental limitations at this site have been documented previously [16]. Accordingly, treatment-related differences observed in the present study should be interpreted as exploratory and context-dependent patterns rather than definitive causal relationships.

Two fertilization treatments were included in this study: (1) NK, receiving synthetic nitrogen (N) and potassium (K) fertilizers only; and (2) NPK, receiving synthetic nitrogen (N), phosphorus (P), and potassium (K) fertilizers. Nitrogen, phosphorus, and potassium were supplied in the form of urea, single superphosphate, and potassium sulfate, respectively. For winter wheat, fertilizer application rates were 165.0 kg N ha^−1^, 57.6 kg P ha^−1^, and 68.5 kg K ha^−1^, while for summer maize the corresponding rates were 187.5 kg N ha^−1^, 24.6 kg P ha^−1^, and 77.8 kg K ha^−1^. All fertilizers were evenly broadcast on the soil surface of the respective plots. For winter wheat, fertilizers were incorporated into the plough layer (approximately 20 cm) using a rotary cultivator 1–3 days before sowing. For summer maize, fertilizers were manually incorporated into the soil to a depth of approximately 10 cm along the crop rows about six weeks after sowing. Winter wheat was typically sown in mid-October and harvested in early June of the following year. Summer maize was sown immediately after wheat harvest and harvested approximately three months later, in late September or early October. The plots were irrigated with groundwater as required, with one to two irrigation events during the winter wheat growing season and two to four events during the summer maize growing season, applying approximately 90 mm of water per irrigation. Aboveground crop residues were removed after harvest, and all plots were conventionally tilled using a rotary tiller.

### 2.2. Sample Collection

Fresh soil samples were collected in 2018, while archived air-dried soil samples preserved for 1, 8, 18, and 28 years were collected in 2017, 2010, 2000, and 1990, respectively. All soil samples were collected immediately after winter wheat harvest in June. Each plot was divided into three equal sections, and ten soil cores (1.5 cm in diameter and 20 cm in depth) were collected from each section and composited to obtain three replicate samples per plot. Fresh soil samples were passed through a 2 mm sieve, transported to the laboratory under cooled conditions in an ice box, and stored at −80 °C prior to DNA extraction. Archived soil samples were naturally air-dried at room temperature after collection and stored in plastic containers under ambient conditions. After sieving through a 2 mm mesh, a subsample of 50 g air-dried soil was adjusted to 60% of field water-holding capacity using sterilized distilled water, placed in Petri dishes, and incubated in the dark at 28 °C for 14 days to obtain rewetted soil samples [17]. Approximately 2 g of fresh, air-dried, and rewetted soil samples were transferred into sterilized 2 mL vials and transported under cooled conditions to Shanghai Personal Biotechnology Co., Ltd. (Shanghai, China), for DNA extraction and high-throughput sequencing.

### 2.3. DNA Extraction, PCR Amplification and Pyrosequencing

Soil DNA was extracted from 0.5 g of soil using the DNeasy PowerSoil Kit (QIAGEN, Hilden, Germany) according to the manufacturer’s instructions. The extracted DNA was stored at −80 °C until further analysis. DNA concentration and purity were determined using a NanoDrop ND-1000 spectrophotometer (Thermo Fisher Scientific, Wilmington, DE, USA), and DNA integrity was evaluated by agarose gel electrophoresis.

PCR amplification of the bacterial 16S rRNA gene was performed using the forward primer 338F (ACTCCTACGGGAGGCAGCA) and the reverse primer 806R (GGACTACHVGGGTWTCTAAT). Each 25 μL reaction contained 1× Q5 Reaction Buffer, 1.25 U of Q5 High-Fidelity DNA Polymerase (New England Biolabs, Ipswich, MA, USA), 200 μM dNTPs, 0.4 μM of each primer, approximately 2 μL of template DNA, and 8.75 µL of nuclease-free water. The thermal cycling conditions included an initial denaturation at 98 °C for 2 min, followed by 25 cycles of denaturation at 98 °C for 15 s, annealing at 55 °C for 30 s, and extension at 72 °C for 30 s, with a final extension at 72 °C for 5 min [18,19]. A plasmid standard curve was constructed as described previously [20]. Serial ten-fold dilutions of the cloned plasmid were used to generate the standard curve, from which copy numbers were derived automatically. The R^2^ value of the amplification efficiency was 0.997. PCR amplicons were purified using Agencourt AMPure Beads (Beckman Coulter, Brea, CA, USA) and quantified with the PicoGreen dsDNA assay kit (Invitrogen, Carlsbad, CA, USA). Following quantification, equimolar amounts of amplicons were pooled and subjected to paired-end sequencing (2 × 300 bp) on the Illumina MiSeq platform (MiSeq Reagent Kit v3) at Shanghai Personal Biotechnology Co., Ltd. (Shanghai, China).

Sequencing data were processed using the Quantitative Insights Into Microbial Ecology pipeline (QIIME, v1.8.0). Raw reads were initially demultiplexed based on exact barcode matches to assign sequences to their corresponding samples. Low-quality sequences were filtered according to the following criteria [20,21]: sequence length <150 bp, average Phred quality score <20, presence of ambiguous bases, or homopolymer runs exceeding 8 bp. Paired-end reads were subsequently merged using FLASH CS6 [22]. After chimera removal, high-quality sequences were clustered into operational taxonomic units (OTUs) at 97% sequence similarity using UCLUST (Edgar 2013) [23]. Representative sequences for each OTU were selected with default parameters and taxonomically assigned by BLAST (https://blast.ncbi.nlm.nih.gov/Blast.cgi, accessed on 10 December 2025) alignment against the Greengenes reference database using a best-hit approach [24]. An OTU table was constructed to summarize the abundance and taxonomic affiliation of OTUs in each sample [25]. OTUs accounting for less than 0.001% of the total sequences were removed to reduce potential sequencing noise [26]. To minimize the influence of uneven sequencing depth among samples, the OTU table was normalized by repeatedly rarefying each sample to 90% of the minimum sequencing depth across all samples for 100 iterations, followed by averaging and rounding of OTU counts. This approach was applied to ensure robustness in downstream comparative analyses. The bioinformatics workflow (QIIME v1.8.0, 97% OTU clustering, and Greengenes database) is an established approach for long-term comparative studies but provides lower taxonomic resolution than modern pipelines (e.g., amplicon sequence variants and updated databases). Consequently, fine-scale differences in community composition and rare taxa should be interpreted with caution and not overemphasized.

### 2.4. Soil Microbial Community Similarity

Bray–Curtis dissimilarity was calculated to quantify differences in microbial community composition using the vegan package in R 4.42 [27,28]. Bray–Curtis similarity was derived as:

(1) Similarity = 1 − dissimilarity

Community similarity was calculated after square-root transformation of the similarity values obtained using Equation (1), following the approach described by Wang et al. [10]. Bray–Curtis similarities between archived air-dried and rewetted soils (1990, 2000, 2010, and 2017) and fresh soil (2018) were computed. This resulted in 72 similarity values for each treatment, corresponding to eight temporal comparisons and nine replicate samples. Bray–Curtis similarity was used as a descriptive metric to illustrate temporal trajectories in microbial community composition rather than for formal hypothesis testing.

### 2.5. Statistical Analyses

Statistical analyses were performed using IBM SPSS Statistics version 21. Due to pseudoreplication, formal inference from ANOVA/LSD between fertilization treatments is not appropriate; comparisons are presented descriptively. Bray–Curtis dissimilarity calculations, Spearman correlation analyses, and boxplot visualizations were conducted using the vegan package in R [27,28].

## 3. Results

### 3.1. DNA-Based Bacterial Community Composition of Air-Dried and Rewetted Soils Archived for Various Time Spans

**Figure 1:**
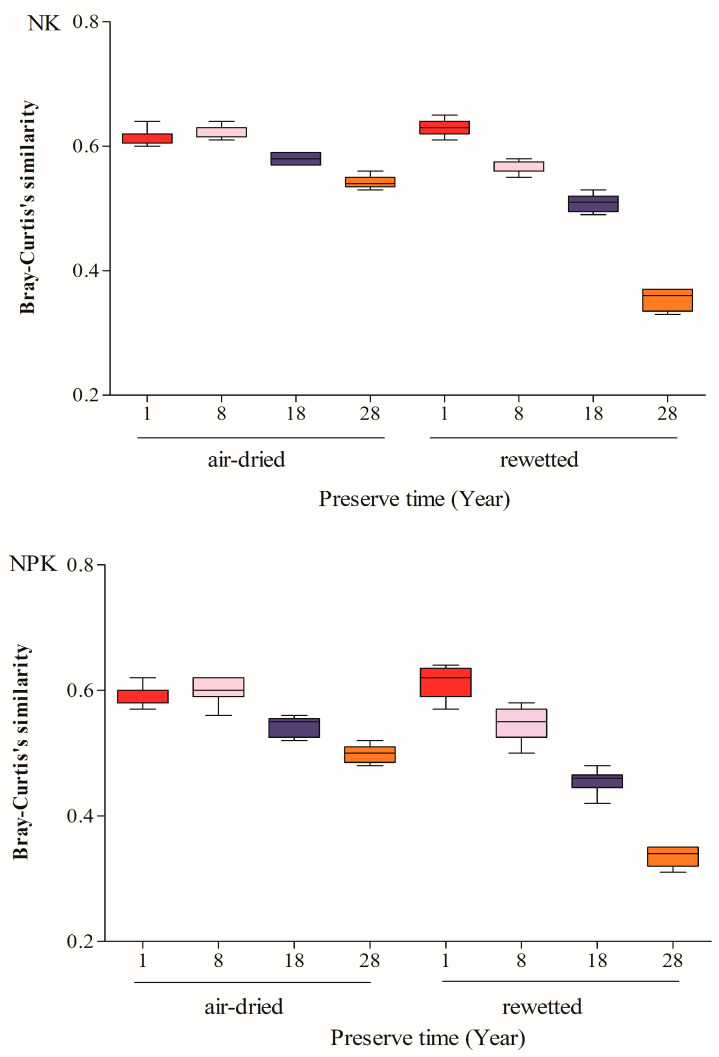
Comparison of Bray–Curtis similarity, with fresh soil as reference, of the microbial community between the archived air-dried and rewetted soils preserved for a different number of years and treated with synthetic NK and NPK fertilizers. Boxes with same color represent the same soil sample. The upper and lower limits of each box represent the 25th and 75th percentiles of parameter values, respectively. The horizontal lines in the center of the box indicate the median values while those outside the box indicate the maximum and minimum values.

The similarity of DNA-based bacterial community composition between archived air-dried soils and their corresponding rewetted soils, under both NK and NPK treatments, decreased with increasing storage duration when compared with fresh soils, following a quadratic relationship (Figure 1 and Figure S1). The coefficients of determination (R^2^) for the quadratic regressions of air-dried soils under NK and NPK treatments were 0.43 and 0.49, respectively, whereas higher R^2^ values were observed for rewetted soils (0.73 for NK and 0.76 for NPK).

Relative to fresh soils, the similarity of bacterial community composition in air-dried soils archived for 1 and 8 years under NK and NPK treatments decreased by an average of 26% and 25%, respectively. In contrast, air-dried soils archived for 18 and 28 years exhibited greater reductions in similarity, averaging 31% and 36%, respectively, which were lower than those observed for soils archived for shorter durations. Similarly, the similarity of DNA-based bacterial community composition in rewetted soils declined progressively with increasing storage time, with reductions of 24%, 32%, 41%, and 58% for soils archived for 1, 8, 18, and 28 years, respectively, compared with fresh soils. Notably, the decrease in similarity was more pronounced in rewetted soils than in the corresponding air-dried soils across all storage durations (Figure 1 and Figure S1).

**Figure 2:**
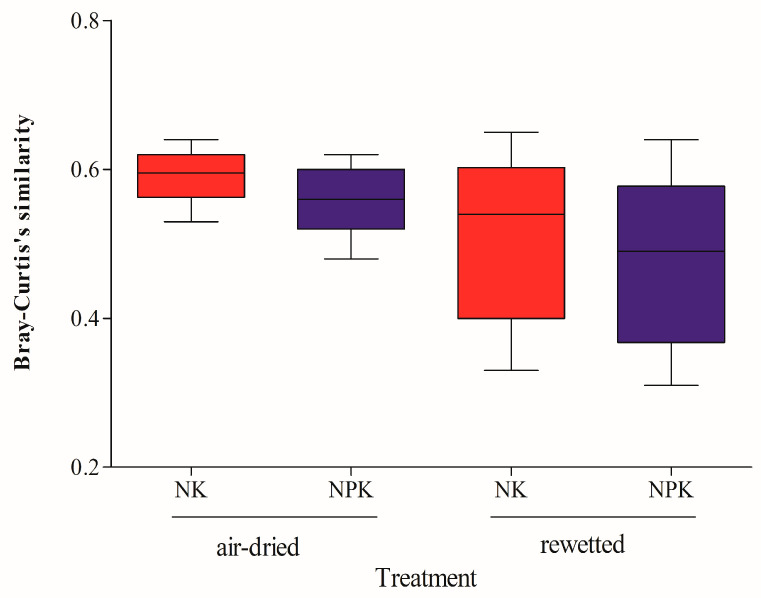
The Bray–Curtis similarity, with fresh soil as reference, of the microbial community showing the variations between the archived air-dried and rewetted soils, treated with synthetic NK and NPK fertilizers, across preservation years. Boxes with the same color represent the same fertilization treatment across preservation years. The upper and lower limits of each box represent the 25th and 75th percentiles of parameter values, respectively. The horizontal lines in the center of the box indicate the median values. The dotted lines indicate the mean values while those outside the box indicate the maximum and minimum values.

No apparent differences in bacterial community composition similarity were observed between the NK and NPK fertilization regimes in this experiment (Figure 2). However, with increasing storage duration, the decline in similarity appeared somewhat greater under NPK than NK (descriptive observation; Figure S1).

### 3.2. The Relative Abundance of Bacteria in Fresh Soil, Archived Soil and Rewetted Soil at the Phylum Level

**Figure 3:**
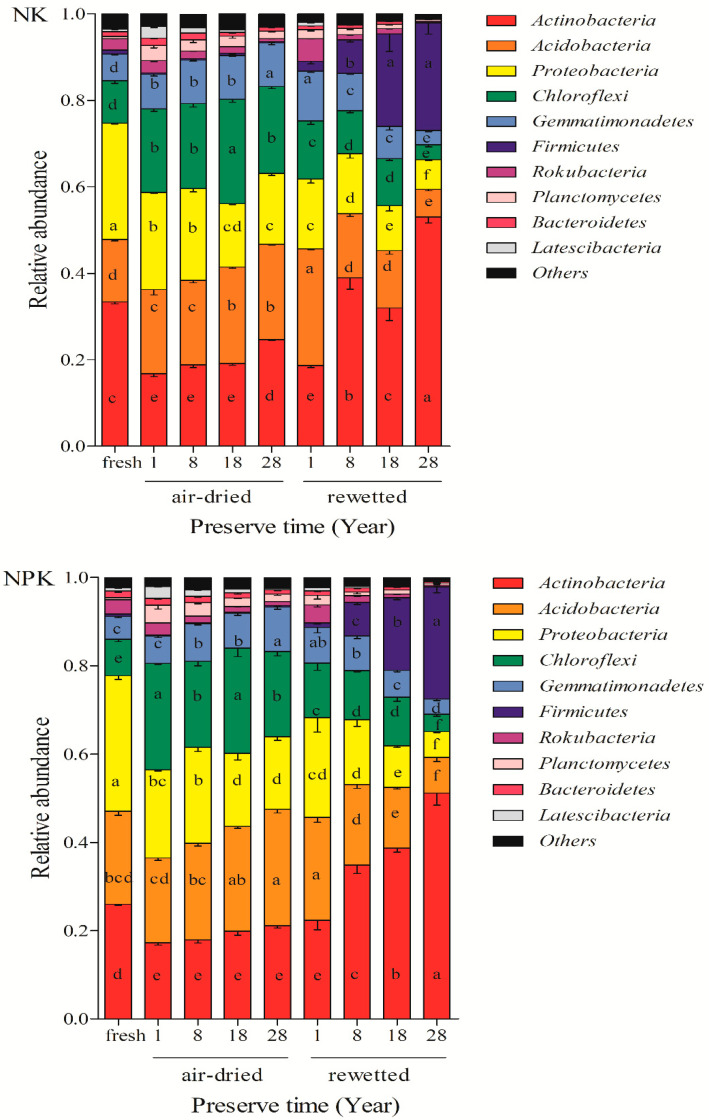
Relative abundances of different phyla of soil bacteria expressed as a percentage of the total community of the fresh soils, archived air-dried soils, and rewetted soils preserved for different timescales, treated with synthetic NK (upper panel) and NPK (lower panel) fertilizers. Note: Data presented are treatment means (n = 3). Different lowercase letters for the same phylum (color) indicate significant differences (*p* < 0.05) among soil samples within the same fertilization treatment. Due to pseudoreplication (single plot per fertilization treatment), no formal statistical comparisons were performed between NK and NPK treatments; differences between fertilization regimes are presented descriptively only.

To evaluate the effects of air-drying and rewetting on bacterial community composition at the phylum level, the relative abundances of the ten dominant bacterial phyla were analyzed in air-dried soils archived for different durations under NK and NPK treatments and their corresponding rewetted soils. Across all treatments and storage durations, the same ten dominant phyla were detected in both air-dried and rewetted soils as in fresh soils (Figure 3 and Figure S2).

Six of the ten dominant phyla exhibited similar temporal trends in relative abundance in rewetted soils as in the corresponding air-dried soils. Specifically, *Actinobacteria*, *Proteobacteria*, *Rokubacteria*, *Planctomycetes*, *Bacteroidetes*, and *Latescibacteria* displayed consistent trends, with only *Actinobacteria* showing an increase in relative abundance over storage time, while the other five phyla decreased. In contrast, rewetting significantly altered the temporal trends of the remaining four dominant phyla. The relative abundances of *Acidobacteria* and *Gemmatimonadetes* increased with storage time in air-dried soils but decreased following rewetting. *Chloroflexi* and *Firmicutes* exhibited stable relative abundances in air-dried soils; however, after rewetting, *Chloroflexi* decreased markedly, whereas *Firmicutes* increased (Figure S2).

Across all storage durations, the mean relative abundances of Actinobacteria and Firmicutes in rewetted soils appeared higher than in the corresponding air-dried soils. *Rokubacteria* showed little difference between air-dried and rewetted conditions. For the remaining dominant phyla, mean relative abundances appeared higher in air-dried than rewetted soils (descriptive patterns; Figure 3; Table S1).

### 3.3. DNA-Based Community Structure of Air-Dried Soils and Their Rewetted Soils

**Figure 4:**
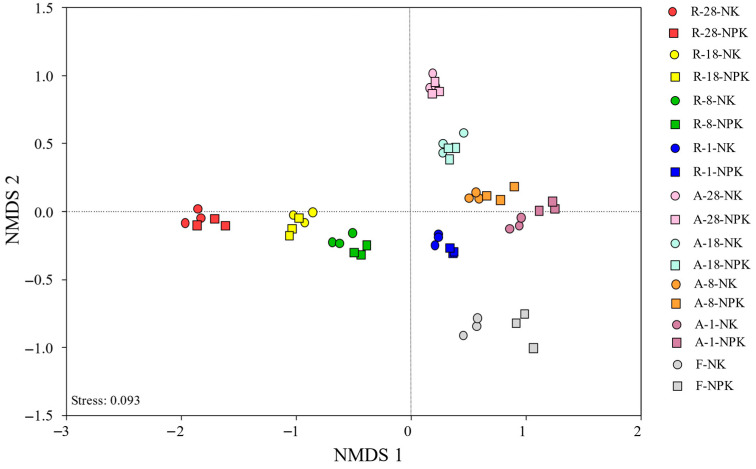
Non-metric multidimensional scaling analysis (NMDS) of overall microbial DNA-based community structure of fresh soils, archived air-dried soils, and rewetted soils preserved for different timescales, treated with synthetic NK and NPK fertilizers. Note: A stands for air-dried soil, F denotes fresh soil, and R refers to rewetted soil. Different colors represent different years of preservation of soil samples; solid circles and solid squares represent NK and NPK treatments, respectively. The stress value indicates the unexplained part of the NMDS.

**Figure 5:**
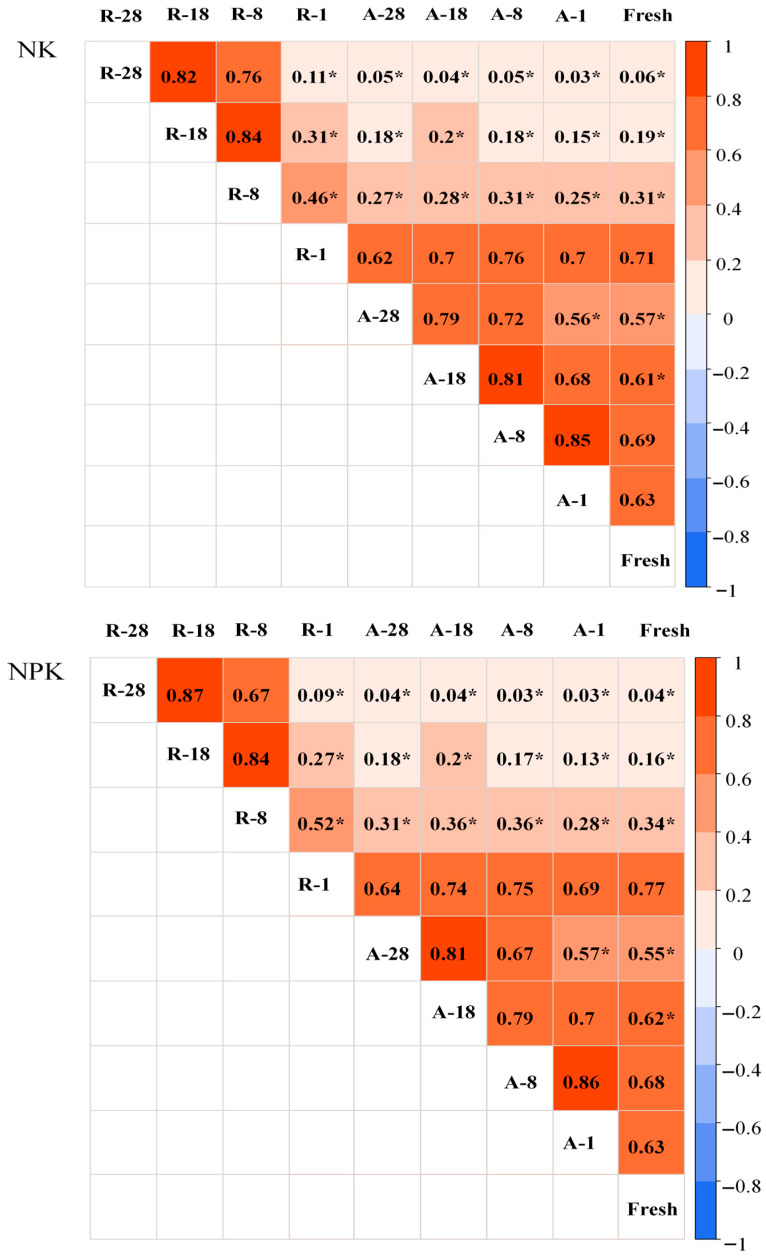
Spearman correlation coefficient represented by color gradient for pairwise comparison of soil microbial community profiles of the different soil samples. Note: A stands for air-dried soil, Fresh means fresh soil, and R refers to rewetted soil. The number linked to A or R with a dash indicates the preservation years of the soil sample. * represents significant difference in soil microbial community profiles between the corresponding soil samples at *p* < 0.05 probability level.

The DNA-based community structures of the air-dried soils which had been archived for 1 year and 8 years and the rewetted soil archived for 1 year were the closest to that of the fresh soils (Figure 4). The Spearman correlation coefficient also showed that there was no significant difference in DNA-based community structure between the soils stated above (Figure 5). In addition, the DNA-based community structure of air-dried soils which had been archived for 18 and 28 years and rewetted soils which had been archived for 8 years or more was significantly different from that of freshly collected soils (Figure 5).

## 4. Discussion

### 4.1. Preservation of DNA-Based Bacterial Community Profiles in Air-Dried and Rewetted Soils

Our results showed no apparent differences in DNA-based bacterial community composition (Figure 1) and structure (Figure 4 and Figure 5) between fresh soils and those air-dried or rewetted after short-term storage (≤8 years), indicating minimal loss of microbial information during this period. For soils archived for only 1 year, rewetting had little effect, with similarities comparable to air-dried counterparts. These patterns are consistent with previous observations that short-term air-drying and storage preserve bacterial community profiles effectively [9,10,29].

With increasing storage duration, similarity to fresh soils declined in both air-dried and rewetted samples, following a quadratic trend, but the decline appeared more pronounced in rewetted soils (Figure 1 and Figure S1). Rapid moisture depletion during air-drying helps prevent DNA degradation [30], and certain bacteria tolerate desiccation through spore/cyst formation or adsorption to soil particles [31,32,33,34,35,36,37,38]. Nevertheless, gradual losses occur due to cell lysis, DNase activity, photolysis, and oxidative processes [39,40,41,42,43,44].

Rewetting introduces additional selectivity. Bacteria differ in drought tolerance [45]; resilient taxa may dominate upon rehydration, potentially suppressing others [45,46,47]. Microbial responses to moisture also vary, with some classified as rapid, intermediate, or delayed responders [48]; our 14-day incubation may have been insufficient for full recovery of slower taxa. Furthermore, 16S rRNA sequencing captures DNA from living, dead, or extracellular sources [49,50]; air-drying preserves historical fingerprints, whereas rewetting selectively amplifies resilient taxa. These factors, along with comparisons to activity-based studies [9,13], explain the greater preservation in air-dried soils over longer storage.

### 4.2. Exploratory Observations on Patterns Associated with Fertilization Regimes

In this specific long-term experiment, the similarity of DNA-based bacterial community composition to fresh soils appeared to decline somewhat more in NPK-treated soils than in NK-treated soils, on average, for both air-dried and rewetted samples, though differences were not substantial (descriptive site-specific patterns; Figure 2 and Figure S1). Clark and Hirsch (2008) [9] similarly reported greater declines in long-term NPK-fertilized soils than in farmyard manure treatments, attributing differences to organic matter content. Here, soil organic matter was higher under NPK, suggesting that factors other than total organic carbon may contribute. Any exploratory association with nutrient management remains speculative and cannot be generalized beyond this experimental system due to pseudoreplication and the lack of plot replication.

### 4.3. Changes in Relative Abundance of Dominant Bacterial Phyla

Reports comparing the relative abundance of bacterial phyla in fresh, air-dried, and rewetted soils over different storage durations are limited. In our study, the dominant bacterial phyla remained largely unchanged across fresh, air-dried, and rewetted soils under both NK and NPK treatments (Figure 3). This finding is consistent with previous studies, such as Barnard et al. (2013) [51], who observed stable dominant phyla in grassland soils under drought and rainfall, and Liu et al. (2019) [8], who reported shared dominant phyla in archived soils. The consistency suggests that these bacterial groups are highly resilient across different soil types and storage conditions.

Overall, the DNA-based community structure of rewetted soils differed substantially from that of fresh soil compared with air-dried soils, likely due to pronounced shifts in the relative abundance of dominant bacterial phyla (sum of relative abundance >95%). For instance, the combined relative abundance of Actinobacteria and Firmicutes increased moderately in air-dried soils (from 17% to 23%) over the storage period, but rose dramatically in rewetted soils (from 21% to 77%). Such changes contributed to the relative decline of other phyla.

This pattern can be explained by the intrinsic traits of these dominant groups. Firmicutes, as typical spore-forming bacteria, rapidly form spores under drought stress [9], and rehydration promotes spore germination and growth [52]. Similarly, Actinobacteria possess strong drought resistance and spore-forming ability, allowing them to maintain viability during storage and proliferate after rewetting [53,54]. In contrast, the relative abundances of other dominant phyla, including *Acidobacteria*, *Gemmatimonadetes*, *Chloroflexi*, and *Proteobacteria*, generally decreased after rewetting regardless of their trends in air-dried soils. For example, in air-dried soils, these phyla ranged from 19 to 24% (*Acidobacteria*), 7.1–10.1% (*Gemmatimonadetes*), 20–24% (*Chloroflexi*), and 16–21% (*Proteobacteria*), whereas in rewetted soils, they decreased to 7–27%, 3.4–10.2%, 4–14%, and 6–17%, respectively.

The decline of these phyla in rewetted soils may result from several factors: (1) slower-growing taxa like *Acidobacteria* fail to recover during short-term rehydration [55,56]; (2) *Gemmatimonadetes* thrive in dry environments but are poorly adapted to humid conditions [57,58,59,60,61,62]; (3) Proteobacteria and *Chloroflexi* exhibit limited stress tolerance or require favorable conditions, with only spore-forming members surviving [63,64]; and (4) the dramatic increase in Actinobacteria and Firmicutes likely suppresses others through competitive interactions. Comparisons with Liu et al. (2019) [8] indicate that trends are influenced by survival strategies, soil properties, and initial abundances. These site-specific patterns highlight differential resilience among phyla and further suggest that air-dried archives better preserve historical DNA-based profiles than rewetted soils.

## 5. Conclusions

This study provides an exploratory assessment of DNA-based bacterial community profiles in long-term archived air-dried and rewetted soils from a single experiment on calcareous loess soil. Because rehydration represents a selective incubation process that may obscure historical community signals, air-dried soils preserved for extended periods can serve as valuable resources for retrospective microbial investigations, particularly those stored ≤8 years, and may better preserve historical DNA-based bacterial profiles than rewetted counterparts, within the limitations of the study design and analytical methods. Both air-drying and rewetting led to some information loss that increased with storage time, but short-term archived soils (≤8 years) retained high similarity to fresh soils.

## Data Availability

The original contributions presented in this study are included in the article/Supplementary Materials. Further inquiries can be directed to the corresponding author.

## Supplementary Materials

The following supporting information can be downloaded at: https://www.mdpi.com/article/10.3390/microorganisms14030595/s1; Table S1: Mean relative abundance (%) of dominant bacterial phyla in air-dried and rewetted soils (averaged across preservation durations of 1, 8, 18 and 28 years) under NK and NPK treatments; Figure S1: The Bray–Curtis similarity of the microbial community between the archived air-dried (a and c) and rewetted soils (b and d) preserved for a different number of years and treated with synthetic NK and NPK fertilizers; Figure S2: Relative abundances of different phyla of soil bacteria expressed as a percentage of the total community of the archived air-dried, rewetted soils treated with NK and NPK fertilizers, respectively, preserved for different timescales.
