# Supplementary material for: DNA-Based Bacterial Community Profiles in Air-Dried Historical Soil Archives Are More Representative than Those from Rewetted Soils
Source: Microorganisms. 2026 Mar 6;14(3):595. doi: 10.3390/microorganisms14030595 (PMC13029469; doi:10.3390/microorganisms14030595)
Supplement: Supplementary file 1 [file microorganisms-14-00595-s001.zip › microorganisms-4067109-supplementary.pdf]

DNA-Based Bacterial Community Profiles in Air-Dried Historical Soil Archives Are More Representative than Those from Rewetted Soils

Peng Lu [1,2,3] , Bingjie Ji [2], Yuan Yan [2], Shulan Zhang [2,3] and Xueyun Yang [2,3,*]

1 Shijiazhuang Institute of Pomology, Hebei Academy of Agricultural and Forestry Sciences, Shijiazhuang 050061, China; lupeng8602@163.com

2 College of Natural Resources and Environment, Northwest A&F University, Yangling, Xianyang 712100, China; jibingjie2707@163.com (B.J.); 18812670037@163.com (Y.Y.); zhangshulan@nwafu.edu.cn (S.Z.)

3 Beijing Changping Soil Quality National Observation and Research Station, Changping, Beijing 100081, China

* Correspondence: xueyunyang@nwafu.edu.cn; Tel.: +86-29-87080050

Table S1 Mean relative abundance (%) of dominant bacterial phyla in air-dried and rewetted soils (averaged across preservation durations of 1, 8, 18 and 28 years) under NK and NPK treatments.

Phylum	Air-dried		Rewetted	
	NK	NPK	NK	NPK
Actinobacteria	0.199 B	0.190 B	0.356 A	0.363 A
Acidobacteria	0.208 A	0.228 A	0.154 B	0.165 B
Proteobacteria	0.187 A	0.187 A	0.118 B	0.120 B
Chloroflexi	0.208 A	0.217 A	0.094 B	0.102 B
Gemmatimonadetes	0.095 A	0.082 A	0.077 A	0.066 B
Firmicutes	0.004 B	0.002 B	0.141 A	0.127 A
Rokubacteria	0.017 A	0.016 A	0.020 A	0.019 A
Planctomycetes	0.026 A	0.027 A	0.012 B	0.012 B
Bacteroidetes	0.013 A	0.013 A	0.007 B	0.007 B
Latescibacteria	0.012 A	0.014 A	0.003 B	0.004 B

Notes: Values are means across the different archiving durations. Different uppercase letters (A, B) within the same fertilization treatment (NK or NPK column pair) indicate significant differences between air-dried and rewetted soils for the same phylum (LSD test, $P < 0.05$). Due to pseudoreplication (single plot per fertilization treatment), no formal statistical comparisons were performed between NK and NPK treatments. Any apparent differences between the two fertilization regimes are descriptive and exploratory only.

Figure Captions

Figure S1 The Bray-Curtis's similarity of the microbial community between the archived air-dried (a & c) and rewetted soils (b & d) preserved for different years treated with synthetic NK and NPK fertilizers.

Figure S2 Relative abundances of different phylum of soil bacteria expressed as a percentage of the total community of the archived air-dried, rewetted soils treated with NK, NPK fertilizers respectively preserved for different time scales.

Figure S1

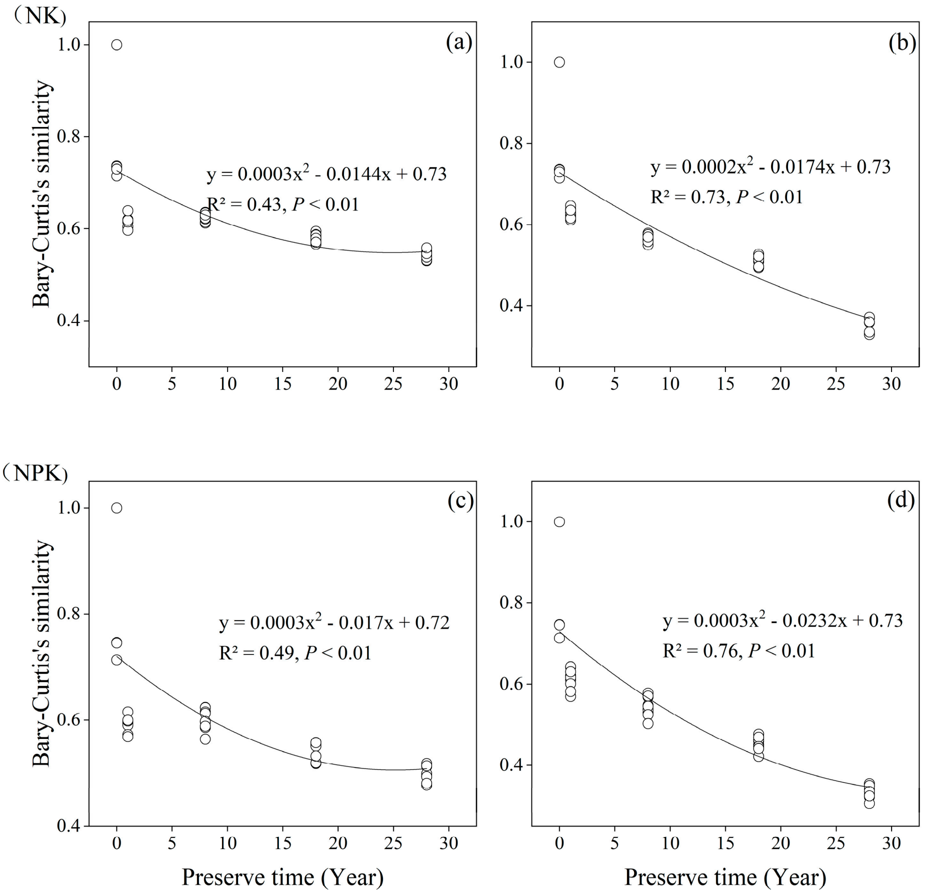

（NK）
（NPK）
(a)
(b)
(c)
(d)
y = 0.0003x2 - 0.0144x + 0.73
R² = 0.43, P < 0.01
y = 0.0002x2 - 0.0174x + 0.73
R² = 0.73, P < 0.01
y = 0.0003x2 - 0.017x + 0.72
R² = 0.49, P < 0.01
y = 0.0003x2 - 0.0232x + 0.73
R² = 0.76, P < 0.01
Bary-Curtis's similarity
Preserve time (Year)
0 5 10 15 20 25 30
0.4 0.6 0.8 1.0

Figure S2

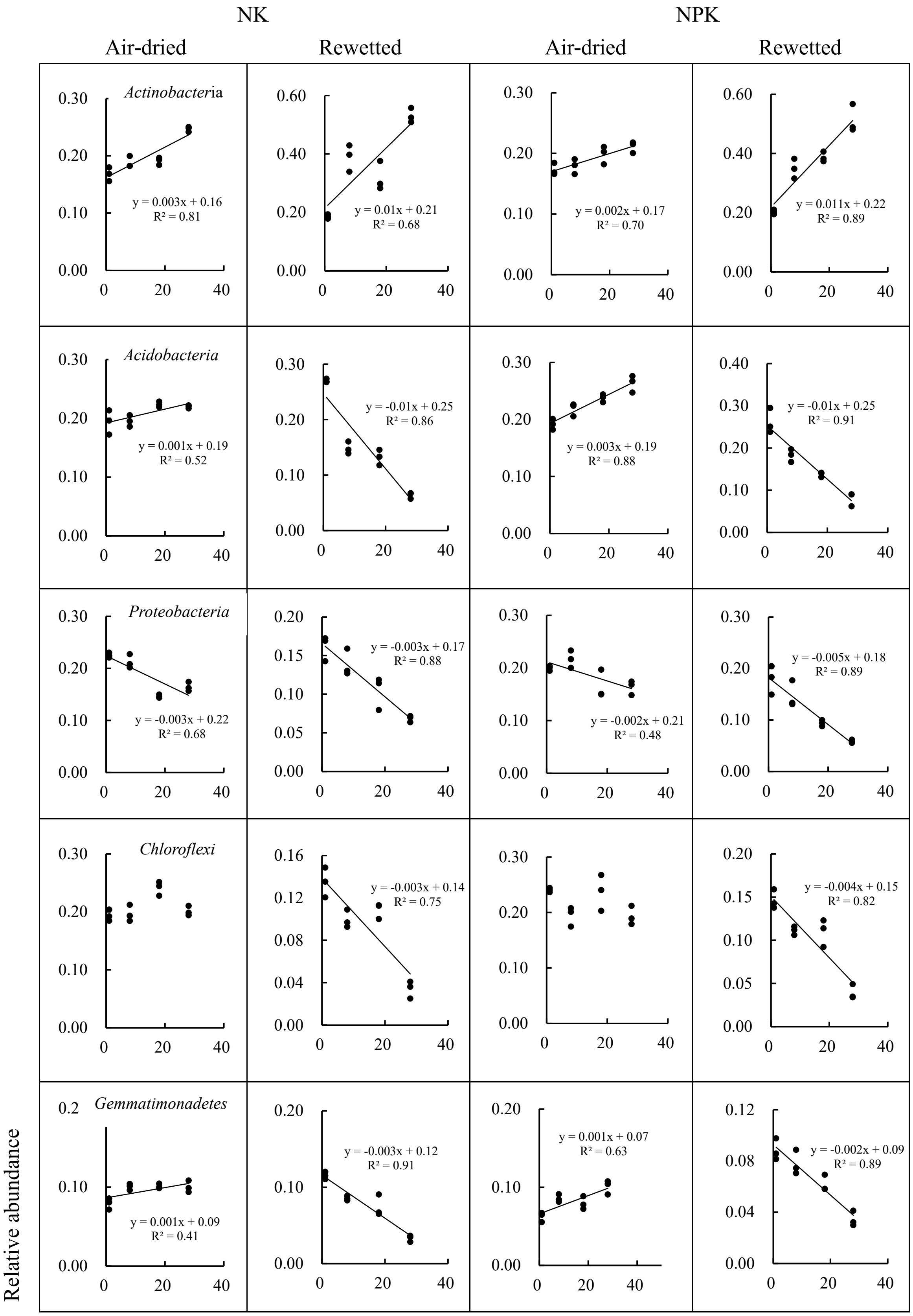
NK
NPK
Air-dried
Rewetted
Air-dried
Rewetted
Actinobacteria
y = 0.003x + 0.16
R² = 0.81
y = 0.01x + 0.21
R² = 0.68
y = 0.002x + 0.17
R² = 0.70
y = 0.011x + 0.22
R² = 0.89
Acidobacteria
y = 0.001x + 0.19
R² = 0.52
y = -0.01x + 0.25
R² = 0.86
y = 0.003x + 0.19
R² = 0.88
y = -0.01x + 0.25
R² = 0.91
Proteobacteria
y = -0.003x + 0.22
R² = 0.68
y = -0.003x + 0.17
R² = 0.88
y = -0.002x + 0.21
R² = 0.48
y = -0.005x + 0.18
R² = 0.89
Chloroflexi
y = -0.003x + 0.14
R² = 0.75
y = -0.004x + 0.15
R² = 0.82
Gemmatimonadetes
y = 0.001x + 0.09
R² = 0.41
y = -0.003x + 0.12
R² = 0.91
y = 0.001x + 0.07
R² = 0.63
y = -0.002x + 0.09
R² = 0.89
Relative abundance

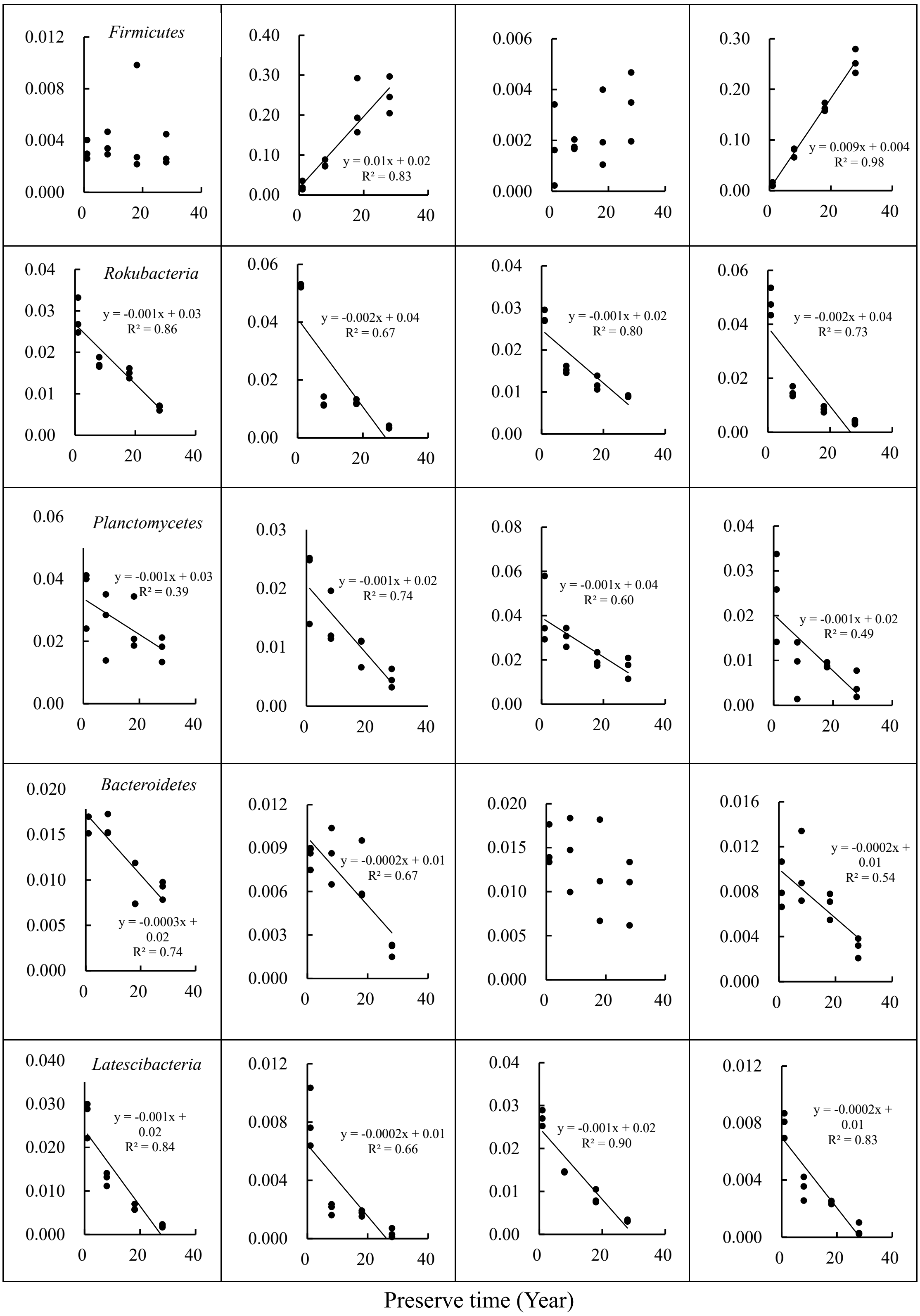

Firmicutes
y = 0.01x + 0.02
R² = 0.83
y = 0.009x + 0.004
R² = 0.98
Rokubacteria
y = -0.001x + 0.03
R² = 0.86
y = -0.002x + 0.04
R² = 0.67
y = -0.001x + 0.02
R² = 0.80
y = -0.002x + 0.04
R² = 0.73
Planctomycetes
y = -0.001x + 0.03
R² = 0.39
y = -0.001x + 0.02
R² = 0.74
y = -0.001x + 0.04
R² = 0.60
y = -0.001x + 0.02
R² = 0.49
Bacteroidetes
y = -0.0003x + 0.02
R² = 0.74
y = -0.0002x + 0.01
R² = 0.67
y = -0.0002x + 0.01
R² = 0.54
Latescibacteria
y = -0.001x + 0.02
R² = 0.84
y = -0.0002x + 0.01
R² = 0.66
y = -0.001x + 0.02
R² = 0.90
y = -0.0002x + 0.01
R² = 0.83
Preserve time (Year)
